# CD44 is a prognostic biomarker and correlated with immune infiltrates in gastric cancer

**DOI:** 10.1186/s12920-022-01383-w

**Published:** 2022-10-31

**Authors:** Weiyan Hou, Lingwei Kong, Zhiping Hou, Hairu Ji

**Affiliations:** 1 College of Basic Medicine, Chengde Medical University, Chengde, China; 2 Department of Orthopaedics, The Affiliated Hospital of Chengde Medical University, Chengde, China; 3 Department of Pathology, Chengde Medical University, Shangerdaohezi Avenue, Chengde, 067000 Hebei China

**Keywords:** CD44, Gastric cancer, Prognosis, Immune infiltration, Bioinformatics

## Abstract

**Objective:**

Gastric carcinoma is the most common malignant tumour of the human digestive system worldwide. CD44 serves as a marker for several tumour stem cells, including gastric cancer. However, the prognostic value of CD44 and its correlation with immune infiltration in gastric cancer remain unclear.

**Methods:**

The relative expression level of CD44 RNA in gastric cancer was analysed in the TCGA and GEPIA2 databases and validated in the GEO database. Differences in CD44 between gastric cancer cell lines and normal cells were detected by real-time PCR, and the HPA database was used to analyse the differential expression of CD44 protein in gastric cancer and normal tissues. The effect of CD44 on the proliferation and migration of gastric cancer cells was detected by CCK8 and transwell assays. UALCAN was used to analyse the relationship between CD44 expression and clinical parameters, and the Kaplan‒Meier Plotter was used to evaluate the prognostic value, including overall survival (OS), progression-free survival (PFS) and post-progression survival (PPS). The CD44 gene and protein interaction network was constructed by using the Linked Omics, GeneMANIA, STRING and DisGeNET databases. GO and KEGG analyses and GSEA of CD44 were performed by using R language. The correlation between CD44 and immune infiltration was explored by using the TIMER, CIBERSORT and GEPIA databases.

**Results:**

CD44 is highly expressed in gastric cancer compared with normal tissues. Inhibition of proliferation and migration of gastric cancer cells after CD44 knockdown was observed. The UALCAN database showed that CD44 was independent of sex in gastric cancer but correlated with cancer stage and lymph node metastasis. Kaplan‒Meier Plotter online analysis showed that OS, PFS and PPS were prolonged in the CD44 low-expression group. GO and KEGG analyses and GSEA results showed that CD44 was mainly located in the endoplasmic reticulum and the extracellular matrix containing collagen, which was mainly involved in protein digestion and absorption. TIMER, CIBERSORT and GEPIA showed that CD44 was associated with infiltrating immune cells and thereby affected survival prognosis.

**Conclusion:**

CD44 is highly expressed in gastric cancer and is an independent prognostic factor associated with immune invasion, which can be used as a candidate prognostic biomarker to determine the prognosis associated with gastric immune invasion.

## Background

Gastric carcinoma is the most common malignant tumour of the human digestive system worldwide. However, as research continues, the incidence and mortality of gastric carcinoma have decreased. Nevertheless, according to the International Agency for Research on Cancer, the incidence of gastric carcinoma was the fifth highest at 5.6%, and the mortality rate was the fourth highest at 7.7% in 2020 [1]. Due to chronic *Helicobacter pylori* infection, life stress and dietary habit changes, the incidence of gastric carcinoma tends to occur in younger people. It is still a major disease affecting human life and health. Currently, the treatment of gastric carcinoma is based on surgery and neoadjuvant therapy, postoperative chemotherapy and biological immunotherapy[2]. However, patients with early gastric cancer generally do not have obvious symptoms, so it is not easy to detect, and most patients diagnosed for the first time already have advanced gastric cancer [3]. Therefore, actively exploring markers of gastric cancer, improving its early screening level and finding targeted factors are the keys to improving the survival rate and prognosis of patients.

CD44, located on human chromosome 11, is an adhesion molecule found on the surface of cells that acts as a receptor for hyaluronic acid, osteopontin, collagen and metalloproteinases [4]. Studies have shown that targeting siCD44 antigen may provide an alternative therapeutic strategy for breast cancer treatment by modulating the IL-1β-mediated inflammatory tumour microenvironment [5]. CD44 is expressed on most circulating tumour cells, whereas matched breast cancer brain metastasis tissues less frequently express CD44 [6]. Direct or indirect downregulation of CD44 expression can inhibit the proliferation, invasion and metastasis of gastric cancer, suggesting that CD44 may be a valuable therapeutic target for gastric cancer [7–9].

According to the cancer stem cell (CSC) hypothesis and studies of other solid tumours, CSCs are highly tumorigenic and have multidirectional differentiation ability and chemoradiotherapy resistance [10]. They are self-renewing and can produce a large number of heterogeneous tumour cells, which are closely related to the occurrence, recurrence and metastasis of tumours. In 2009, Takaishi found that CD44+ cells could form globular colonies in serum-free medium, and xenograft tumours formed when they were injected into the subcutaneous and gastric walls of SCID mice. The existence of a stem cell subpopulation in human gastric carcinoma cell lines has been demonstrated [11]. Subsequent studies have found that only CD44+ cells can form gastric SCS-like tumours [12–14]. Compared with cells of a gastric cancer cell line, the expression of CD44 mRNA is significantly higher in gastric cancer stem cells (CSC-Gs) [15, 16]. Abundant studies have demonstrated that CD44 serves as a marker for several tumour stem cells, including CSC-Gs [15, 17–19].

Tumour stem cells are subpopulations of undifferentiated cancer cells within the tumour bulk, and their external interaction with the surrounding tumour microenvironment can drive tumour growth by interacting with resident and infiltrating nontumor cells [20]. Recent studies have shown the pivotal role of some major immune cells in driving the expansion of CSCs, which concurrently elicit evasion of the detection and destruction of various immune cells through several distinct mechanisms. For instance, tumour-infiltrating myeloid cells interact with CSCs and constitute potential therapeutic targets for the reprogramming of the tumour microenvironment across multiple cancers [21]. Tissue-infiltrating macrophages are an integral component of adult stem cell niches, raising the possibility that this homeostatic mechanism may be leveraged by CSCs for their maintenance [22]; in addition, dendritic cells (DCs) play an essential role in priming the T cell-mediated antitumour immune response by cross-presenting tumour antigens. Tumorigenic DCs fight against CSCs [23], and a vaccine based on these DCs is in clinical trials [24].

Many previous studies have shown that immune infiltration-related factors are highly correlated with the prognosis of different tumours [25–27], and the number of immune-infiltrating cells, including natural killer (NK) cells [28], DCs [29] and CD4 T cells [30], is also highly correlated with tumour prognosis. CD44 expression is positively correlated with immune cells, and lung adenocarcinoma patients with higher CD44 levels have worse overall survival [31]. The combined index of CD44 and Nanog expression is a promising prognostic predictor of relapse-free survival and overall survival in bladder cancer, which can help determine which patients will benefit from intensive treatment [32]. CD44 is one of the independent prognostic predictors of moderate survival in renal clear cell carcinoma, which may be associated with tumour immunity, but the underlying mechanism remains unclear [33]. In gastric adenocarcinoma, cancer stem cell-like cells are activated, and CD44 is used as a marker in conjunction with MEK to evaluate prognostic indicators of gastric adenocarcinoma patients undergoing gastrectomy [34]. CD44 can be used as an immune checkpoint to evaluate the prognosis of gastric cancer, which can improve the prognostic accuracy of gastric cancer patients [35]. Among a variety of tumour stem cell markers, CD44 is widely involved in immune invasion and is associated with the prognosis of various tumours, including gastric cancer. However, whether its involvement in gastric cancer is related to immune invasion remains unclear.

Therefore, we used The Cancer Genome Atlas (TCGA) database to analyse the relative expression level of CD44 RNA in gastric cancer and validated our findings in the Gene Expression Omnibus (GEO) database. The Human Protein Atlas (HPA) database was used to analyse the differential expression of CD44 protein in gastric cancer and normal tissues. The relationships between CD44 and various clinicopathologies were analysed using the UALCAN database, and the relationships between CD44 and prognosis were plotted using the Kaplan‒Meier Plotter database. We combined Linked Omics, GeneMANIA, the STRING database and bioinformatics related to TCGA, Gene Ontology (GO) and Kyoto Encyclopedia of Genes and Genomes (KEGG) analyses and gene set enrichment analysis (GSEA) to analyse the relationship between CD44-related genes and signalling pathways. Finally, the relationship between CD44 and immune cell markers was analysed using the Tumour Immune Estimation Resource (TIMER) database, CIBERSORT and Gene Expression Profiling Interactive Analysis (GEPIA). The objective was to explore the prognostic value of CD44 in gastric cancer and the possible mechanism of immune invasion.

## Methods

### RNA and protein expression of CD44 in gastric cancer

GEPIA2 (http://gepia2.cancer-pku.cn/#index) is an updated version of GEPIA. It can be used to analyse the RNA expression sequencing data of 9736 tumour samples and 8587 normal samples in TCGA and GTEx. We used this database to analyse the expression of CD44 across cancers. After cross-analysis with the results obtained by using TIMER, the common types of differential cancer were obtained.

The original RNA expression data of 407 gastric cancer cases were downloaded from the TCGA database (http://portal.gbc.cancer.gov/). The TCGA database, the largest database of cancer genetic information, currently includes 33 cancer types based on large-scale genome sequencing, and genomic, transcriptomic, epigenetic, proteomic and other omics data are available.

The GEO database (https://www.ncbi.nlm.nih.gov/geo/) includes gene expression test data submitted by research institutions around the world. We downloaded microarray datasets GSE29272 (tumour tissue 134, normal tissue 134, sequencing platform GPL196) and GSE79973 (tumour tissue 10, normal tissue 10, sequencing platform GPL570) as the research objects.

The HPA database (https://www.proteinatlas.org) is based on proteomics, transcriptomics and systems biology. It provides information on the tissue and cellular distribution of 24,000 human proteins. The protein expression data and clinical information of normal and tumour tissues are also included.

### The relationship between CD44 expression and various clinicopathological features and prognoses

The UALCAN database (http://ualcan.path.uab/index.hrml) is a comprehensive, interactive Web resource. In the TCGA module, after the input of the target genes CD44 and STAD, the correlation between CD44 and clinicopathological parameters (sex, cancer stage, lymph node metastasis) was analysed online.

The Kaplan‒Meier Plotter database (https://kmplot.com/analysis) comprises an evaluation of the impact of 54,675 genes on survival outcomes in 21 cancers. We analysed the prognostic value of CD44 in gastric cancer using the Kaplan‒Meier Plotter database, including overall survival (OS), progression-free survival (PFS) and post-progression survival (PPS).

### Gene networks and gene targets for gastric cancer

The Linked Omics database (http://www.linkedomics.org) is a database of multiple omics and clinical data for 32 cancer types. Data types include miRNA, mRNA data, methylation data, mutation sites, etc. We used this database to obtain heat maps of genes positively and negatively correlated with CD44 in gastric cancer.

The GeneMANIA database (http://genemania.org) enables the prediction of gene function. We used the database’s rich genomics and proteomics to construct a network of genes that interact with CD44 and explored genes with similar functions to CD44.

The STRING database (https://string-db.org/) is a database that analyses protein interactions. The protein–protein interaction (PPI) network of CD44 was constructed by using this database.

DisGeNET (https://www.disgenet.org/) can be used to retrieve the relationship between genes and diseases and between variant genes and diseases. The database systematically integrates data sources from expert-curated repositories, GEAS catalogues, animal models, and scientific literature. By using this database, we obtained disease-related targets related to “malignant neoplasm of stomach”.

### Gene ontology (GO) and Kyoto encyclopedia of gene and genomics (KEGG) analysis

The biological function of CD44 in gastric cancer was analysed by GO and KEGG analyses. GO analysis can define and describe the function of genes and proteins. A standard language with three levels of structure, including biological process (BP), cell composition (CC), and molecular function (MF), has been developed to provide consistent functional descriptions of gene products in various databases. KEGG analysis provides insights into higher-level functions of genes at the molecular level. We performed functional enrichment analysis of CD44-related genes by using the cluster profile package in R software and visualized the results.

### Gene set enrichment analysis (GSEA)

GSEA uses predetermined gene sets and arranges them in descending order by differential expression levels for gene enrichment analysis. Because GSEA uses whole-genome expression profiles obtained by sequencing or chip analyses, there is no need to specify the threshold value of differential genes, so the results obtained are more reliable than those obtained by GO and KEGG analyses.

### Tumour immune estimation resource (TIMER)

The TIMER database (https://cistrome.shinyapps.io/timer/) is a database related to tumour immunity. It includes immunity, exploration and evaluation. We first performed a holistic assessment of CD44 expression in multiple types of cancer. In addition, the correlation between CD44 and immune cell infiltration, such as B cells, CD8+ T cells, CD4+ T cells, neutrophils, macrophages and dendritic cells, was analysed by using the “gene” module. In addition, the correlation module was used to analyse the relationship between CD44 and immune cell markers.

### Correlation analysis of CD44 and immune checkpoints

The GEPIA database (http://gepia.cancer-pku.cn/) integrates TCGA cancer data with CRTx normal tissue data. It analyses the association and correlation between two genes. We used this database to analyse the correlation between CD44 and immune checkpoint genes.

### Cell culture, siRNA interference and transfection

Human gastric cancer cells (SGC7901 and MGC803) and human gastric mucosal epithelial cells (GES-1) were obtained from Prof. Jie-Min Qi of Chengde Medical University and cultured in an RPMI-1640 medium containing 10% foetal bovine serum (FBS). All cells were incubated in an incubator with 5% carbon dioxide at 37 °C.

The cells were transfected with human CD44 siRNA duplex oligo ribonucleotides, with a 5′-CTCTGAGCATCGGATTTGA-3′ targeting sequence, and negative control duplexes (RiboBio, China) and Lipofectamine 3000 (Invitrogen) were used.

### RNA isolation and real-time PCR

For real-time quantitative PCR (qRT‒PCR), total RNA was extracted from cells with TRIzol reagent (Invitrogen, USA) according to the manufacturer’s instructions. cDNA was synthesized with ABScript II RT Master Mix (ABclonal, China). qRT‒PCR analysis was performed using 2 × Universal SYBR Green Fast qPCR Mix (ABclonal, China). Total RNA levels were normalized to those of GAPDH. The primer sequences were as follows:

CD44, forward: 5′-ACCGACAGCACAGACAGAATC-3′;

CD44, reverse: 5′-GTTTGCTCCACCTTCTTGACTC-3′

GAPDH, forward: 5′-CACAAGCAGAGTGCTGAAGGTG-3′; and

GAPDH, reverse: 5′-ACCACCCTGTTGCTGTAGCCAA-3′.

The results were calculated by the 2^− ΔΔCT^ method. The experiment was repeated at least three times.

### Proliferation assay

Gastric cancer cells were seeded into a 96-well plate at a density of 3 × 10^3^ cells/ml after they were transfected with siCD44, and the cells were evaluated at 0, 24, 48, 72 and 96 h. The culture medium was added to 10 μl CCK8 solution and cultured for 1.5 h, and the OD value was measured at 450 nm. The experiment was repeated at least three times.

### Wound healing test

Gastric cancer cells were seeded into a 6-well plate to form a confluent monolayer and scratched with a 200 μl pipette. The damaged monolayer was washed with phosphate-buffered saline (PBS) to remove cell debris. The cells were cultured in a serum-free medium, and digital images of the wound surface were taken with an inverted microscope after 0 h and 24 h. To evaluate the wound area, we imported the wound image into ImageJ software for calculation. The experiment was repeated at least three times.

### Statistical analysis

Statistical analyses were carried out using GraphPad Prism (Version 9.00). R language was used for the different analysis based on the sample data obtained from the TCGA and GEO databases. The volcano map of differential genes in TCGA gastric cancer samples was drawn by using the R software package ggscatter, and |log^2^FoldChange|> 1 was defined as the differentially Upregulated and downregulated genes. The R software package complot was used to analyse the correlation between genes and infiltrating immune cells. The Kaplan‒Meier plots and survival outcome results are displayed with HR and R values based on a log-rank test. Spearman correlation analysis of GEPIA genes was performed. All grouped data in the figures are the means ± SDs. For all tests in this study, a *P* value of < 0.05 was considered statistically significant.

## Results

### CD44 is highly expressed in gastric cancer

**Fig. 1:**
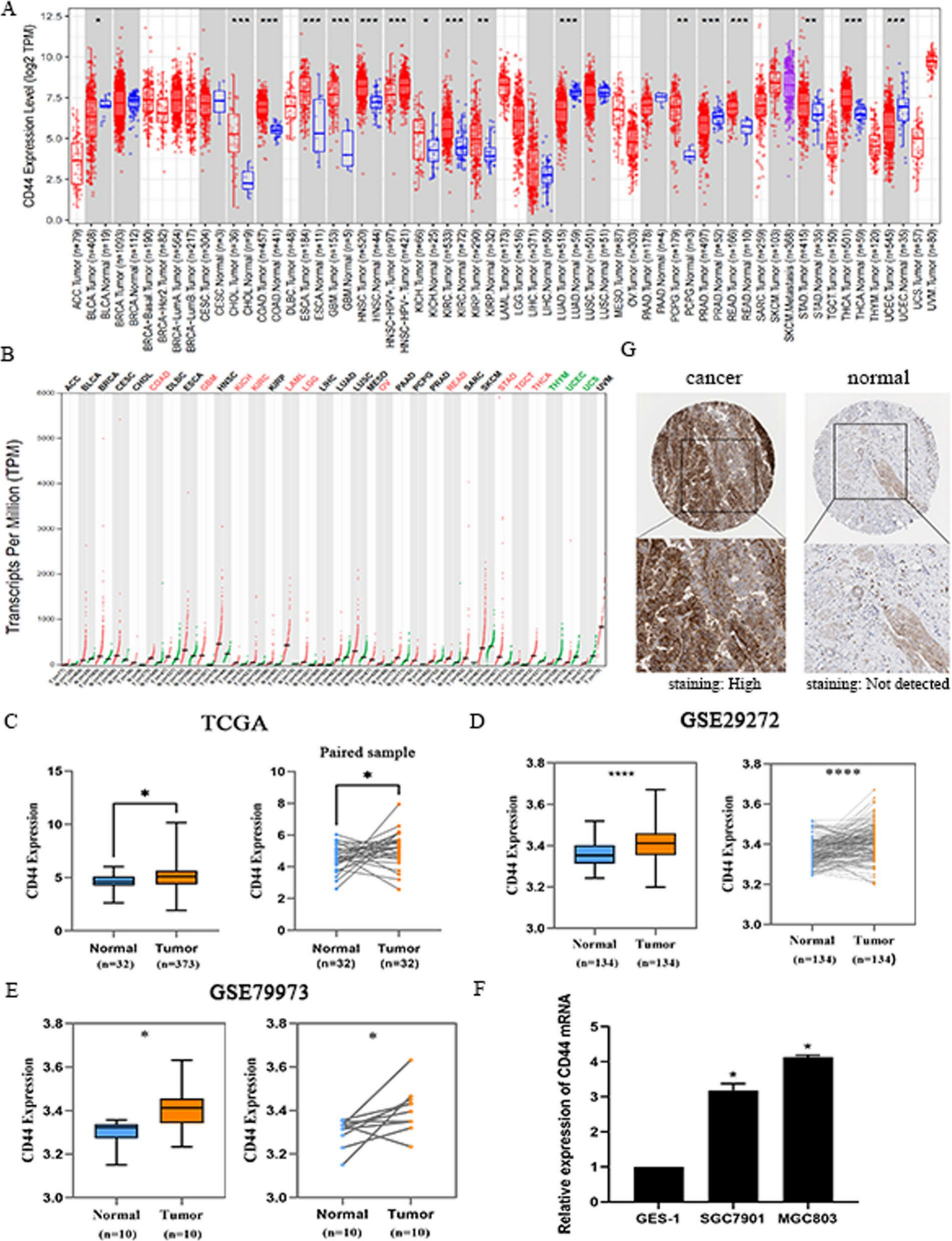
Expression of CD44 in cancer. **A** The TIMER database was used to explore the expression of CD44 in many different cancers. **B** The differential expression of CD44 across cancers was analysed using the GEPIA2 database. **C** Comparison of CD44 expression in normal (n = 32) and tumour (n = 373) tissues in the TCGA database. **D**–**E** In the GEO database, CD44 expression levels in 134 and 10 pairs of gastric adenocarcinoma and normal glands were compared. ****P < 0.0001; *P < 0.05. **F** qRT‒PCR analysis of the relative mRNA expression levels of CD44 in GES-1 normal gastric epithelial cells and 2 types of gastric cancer cells (*P < 0.05). **G** In the HPA database, CAB000316 antibodies were used to detect the dye expression of CD44 protein in gastric cancer patients and normal controls

To evaluate the involvement of CD44 in gastric cancer, we used the Tumour Immune Estimation Resource (TIMER), Gene Expression Profiling Interactive Analysis (GEPIA2), The Cancer Genome Atlas (TCGA), GEO and Human Protein Atlas platform (HPA) to comprehensively analyse whether CD44 exhibits different expression levels of mRNA and protein in gastric cancer. First, an overall evaluation of CD44 expression in a variety of common tumours was made by using the TIMER and GEPIA2 online databases (Fig. 1A–B). After cross-referencing the results from the two databases, the expression of CD44 in 11 cancer species, including COAD, GBM, KICH, KIRC, LAML, LGG, OV, READ, STAD and THCA, was significantly higher than that in normal gastric tissues. CD44 showed low expression levels in YHYM, UCEC and UCS. In addition, according to the analysis of Fig. 1B, the median expression value of CD44 in STAD tumour samples was 177.34, and the median expression value in normal samples was 34.5. In addition, we downloaded the “STAD HTSeq-FPKM (n = 407)” dataset from the TCGA database and divided it into tumour tissue and normal tissue for data collation. Differential analysis of the target gene CD44 was achieved. The results showed that CD44 was highly expressed in the gastric cancer samples (n = 373) compared with the normal adjacent tissue samples (n = 32) and that there were significant differences (*P* = 0.0374 < 0.05) (Fig. 1C). To verify the TCGA results, the differential expression of CD44 mRNA in 134 and 10 pairs of gastric cancer and normal tissue paired samples were analysed using GEO data (GSE29272, GSE79973). It was confirmed that CD44 mRNA was highly expressed in tumour samples (Fig. 1D, E). This is consistent with the results we obtained from mRNA extraction in the gastric cancer cells (Fig. 1F).

To further verify the CD44 transcription profile, staining and expression data from tumour pathological sections obtained from HPA were used. CD44 expression in tumours was compared with that in normal tissues at the same site. Images of immunohistochemically stained tissue samples showed that CD44 was almost not expressed in normal tissues with a quantity of < 25% but that it was significantly overexpressed in tumour tissues with a quantity of 75%–25% (Fig. 1G). In conclusion, the analysis results of mRNA and protein levels showed that CD44 expression was upregulated in gastric cancer, which was different from that in normal tissues.

### CD44 promotes proliferation and migration of gastric cancer cells and is associated with a poor prognosis

**Fig. 2:**
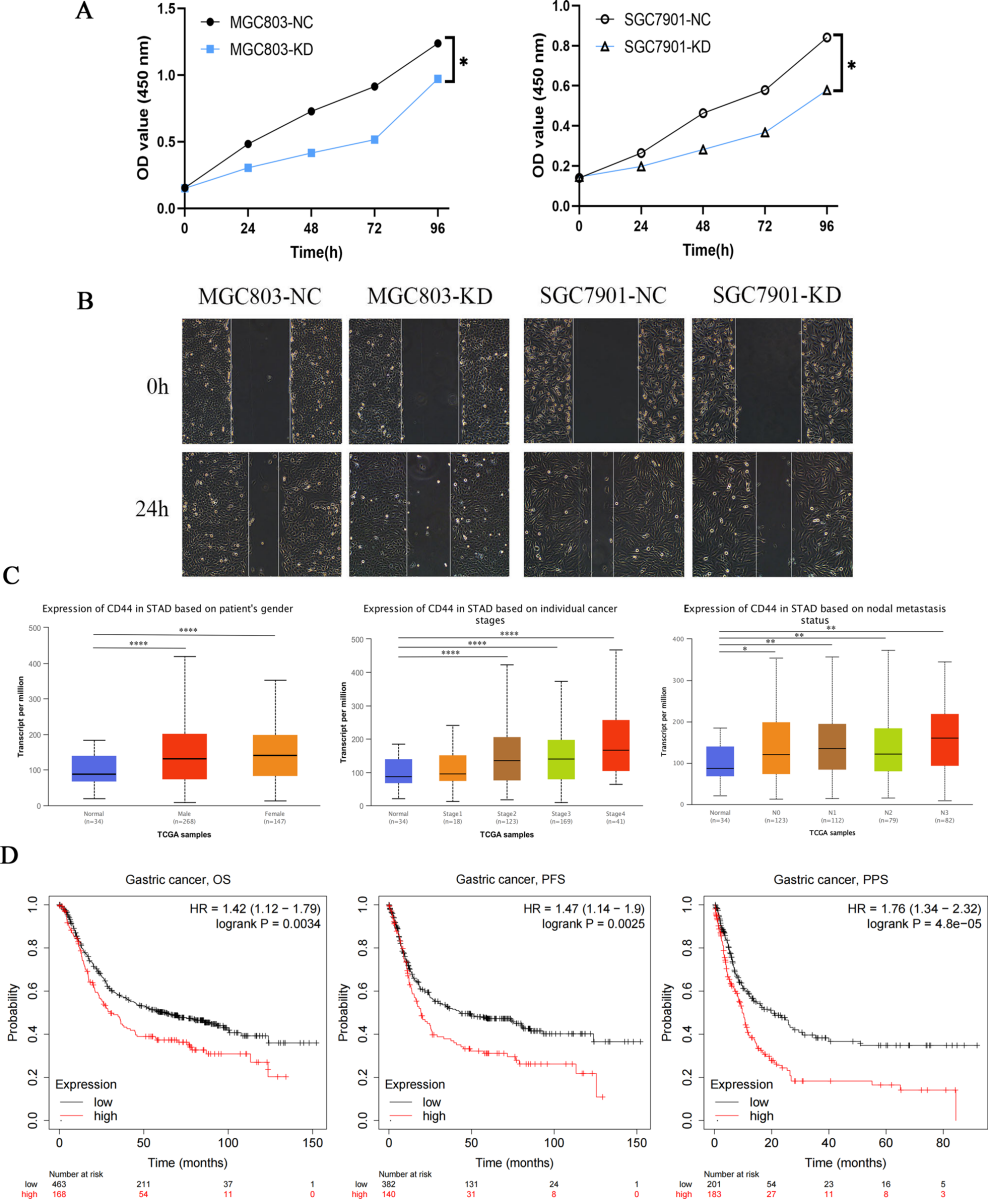
The relationship between clinical parameters and CD44 and the survival curve to evaluate the prognostic value of CD44. **A** CCK8 analysis showed that CD44 knockdown inhibited the proliferation rate of SGC7901 and MGC803 cells (*P < 0.05). **B** The wound healing test showed the migration ability of SGC7901 and MGC803 cells in the case of CD44 knockdown. **C** CD44 expression was evaluated in patient groups with different clinical parameters using the UALCAN database. From left to right, sex, tumour stage, and lymph node metastasis are shown. *P < 0.05, **P < 0.01, ****P < 0.0001. **D** The KM-Plotter database was used to demonstrate the impact of high CD44 expression on the survival and prognosis of OS, PFS and PPS in gastric cancer patients

To determine the role of CD44 in the proliferation and migration of gastric cancer cells, we used siRNA to knock down CD44 in SGC7901 and MGC803 cells. To detect the effect of CD44 knockdown on the proliferation of gastric cancer cells, the results of the CCK8 assay showed that low CD44 expression inhibited the proliferation of SGC7901 and MGC803 cells compared with that in the control group (Fig. 2A). Wound healing analysis was carried out to observe the effect of CD44 on the migration of SGC7901 and MGC803 cells. Knockdown of CD44 expression significantly widened the wound compared with that in the NC group cells (Fig. 2B).

By using the UALCAN database, the expression of CD44 in gastric cancer patients with different clinical parameters was analysed. Based on sex, CD44 expression was significantly increased in both male and female gastric cancer patients compared with the normal control group. However, there was no significant difference in CD44 expression between men and women who had already developed gastric cancer. These results indicated that the expression of CD44 in gastric cancer was independent of the sex of the patients. Based on the analysis of the individual cancer stage, the expression of CD44 was significantly different in stages 2, 3 and 4 (*P* < 0.0001). In terms of nodal metastasis status, compared with that in the normal control group, the expression of CD44 was significantly increased at the N0, N1, N2 and N3 stages. However, there was no difference in the expression of CD44 at the N0, N1, N2 and N3 stages (Fig. 2C). In addition, by reading the literature, it was found that the expression of the CD44 isoform CD44v6 in gastric cancer was related to the location of the tumour, depth of invasion, lymph node metastasis, Lauren classification and TNM stage [36].

Experiments have indicated that gastric cancer patients with high CD44 expression have a high recurrence rate and high metastasis rate, and their prognosis is very poor [37]. CD44 is therefore used as an indicator of poor prognosis in patients. The KM-Plotter database was used on the premise that the target gene was CD44. OS, PFS and PPS were selected in the “Survival” box to obtain the survival analysis curves of the three survival periods. The results showed that the overall survival (OS), progression-free survival (PFS) and post-progression survival (PPS) of gastric cancer patients with high CD44 expression were poor, and all* P* values were less than 0.05 (Fig. 2D). The most significant result was found for PPS. These results further confirmed the correlation between CD44 and the survival prognosis of gastric cancer patients.

### Gene network of CD44 and its presence in the intersection of gastric cancer gene targets

**Fig. 3:**
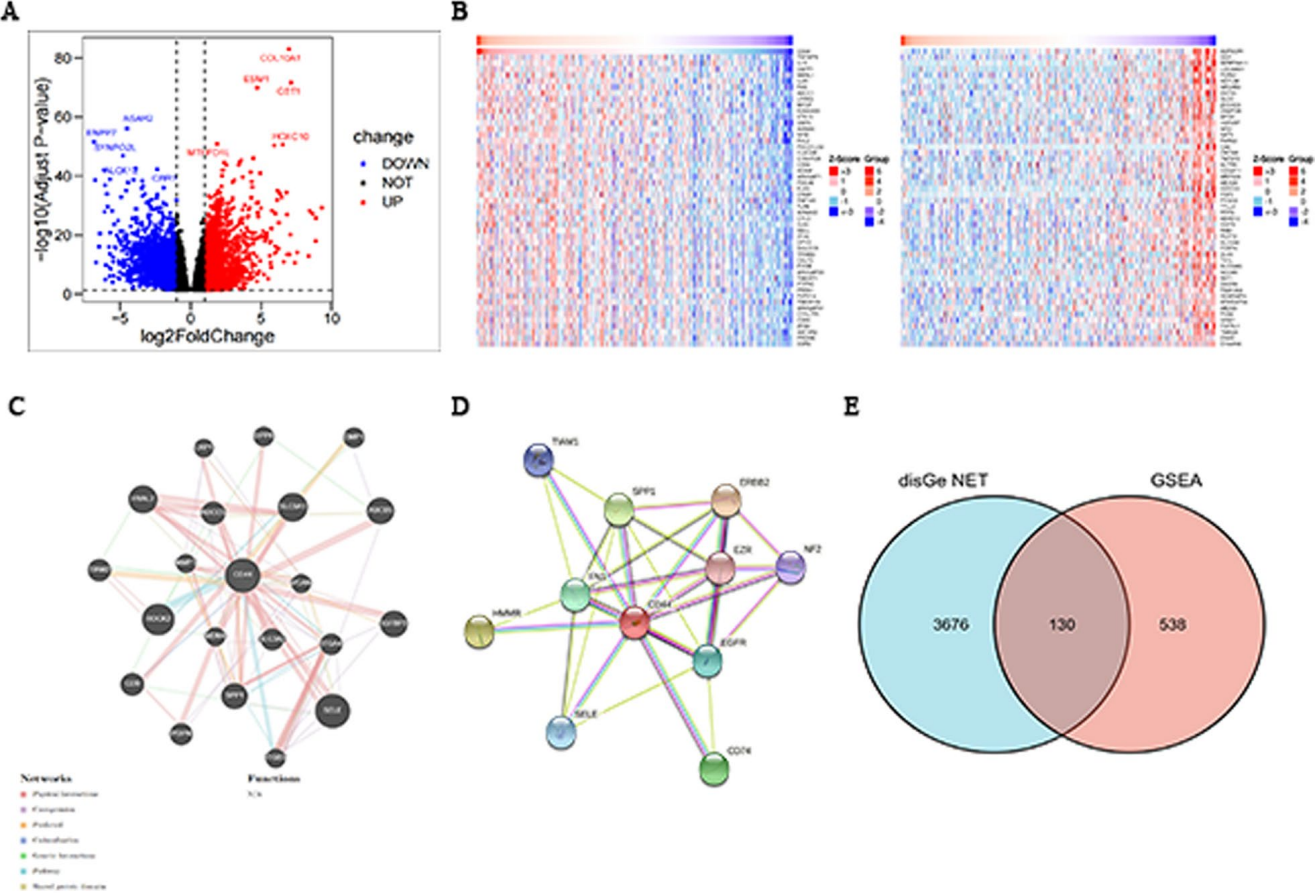
TCGA differential analysis and CD44-related gene analysis. **A** Volcano map based on TCGA gastric cancer data. The red genes are upregulated, the blue genes are downregulated, and the black genes have no change in expression. **B** The heatmap constructed by using the Linked Omics database shows genes that are positively and negatively correlated with CD44 expression. **C** CD44 gene–gene interaction network constructed by using GeneMANIA. **D** The PPI network of CD44 was generated using STRING. **E** Cross-analysis Venn diagram of disease targets from DisGeNET and CD44 differential genes used for GSEA

The volcano map was plotted using the R package ggscatter from the data obtained from TCGA. The differentially expressed genes with log2FoldChange, *P* < 0.05 were read. According to the guidelines, a log2FoldChange > 1 indicates an upregulated gene, and a log2FoldChange < 1 indicates a downregulated gene. The top five upregulated genes and the top five downregulated genes are labelled. According to the volcano plot, there were 2133 upregulated genes, 2349 downregulated genes, and 6836 genes with no significant change in expression (Fig. 3A). The HiSeq RNA database in the TCGA secondary database Linked Omics was used to analyse and predict the correlation between the target gene CD44. The “Pearson correlation test” was selected as the statistical method. The two heatmaps generated indicated genes positively correlated with CD44, including TNFAIP8, IL5, and DAPP1, and genes negatively correlated with CD44, including ASPSCR1, GGH, and SERPINA11 (Fig. 3B).

In addition, the GeneMania database was used to construct a gene–gene network interacting with CD44. The results showed that 20 genes, including SELE, ROCK2, and SLC9A1, were most associated with CD44 (Fig. 3C). A protein–protein interaction network that interacts with CD44 in *Homo sapiens* was obtained using the STRING database. CD44, as the centre, contains 30 edges and 11 nodes. As seen from the figure, genes interacting with CD44 include SELE, SPP1, and EDFR (Fig. 3D). By integrating the results of the two databases above, two genes were found to be identical, namely, SELE and SPP1. This also suggests that further studies should be carried out based on the specific mechanism of their interaction with CD44.

A total of 3806 disease targets of “malignant neoplasm of stomach” were obtained from the DisGeNET database and used for GSEA of CD44 differential cancer. Cross-analysis of the two was used to identify intersections. The results showed that a total of 130 genes overlapped.

### Enrichment analysis of GO and KEGG pathways of CD44 and its related genes in TCGA gastric cancer samples

**Fig. 4:**
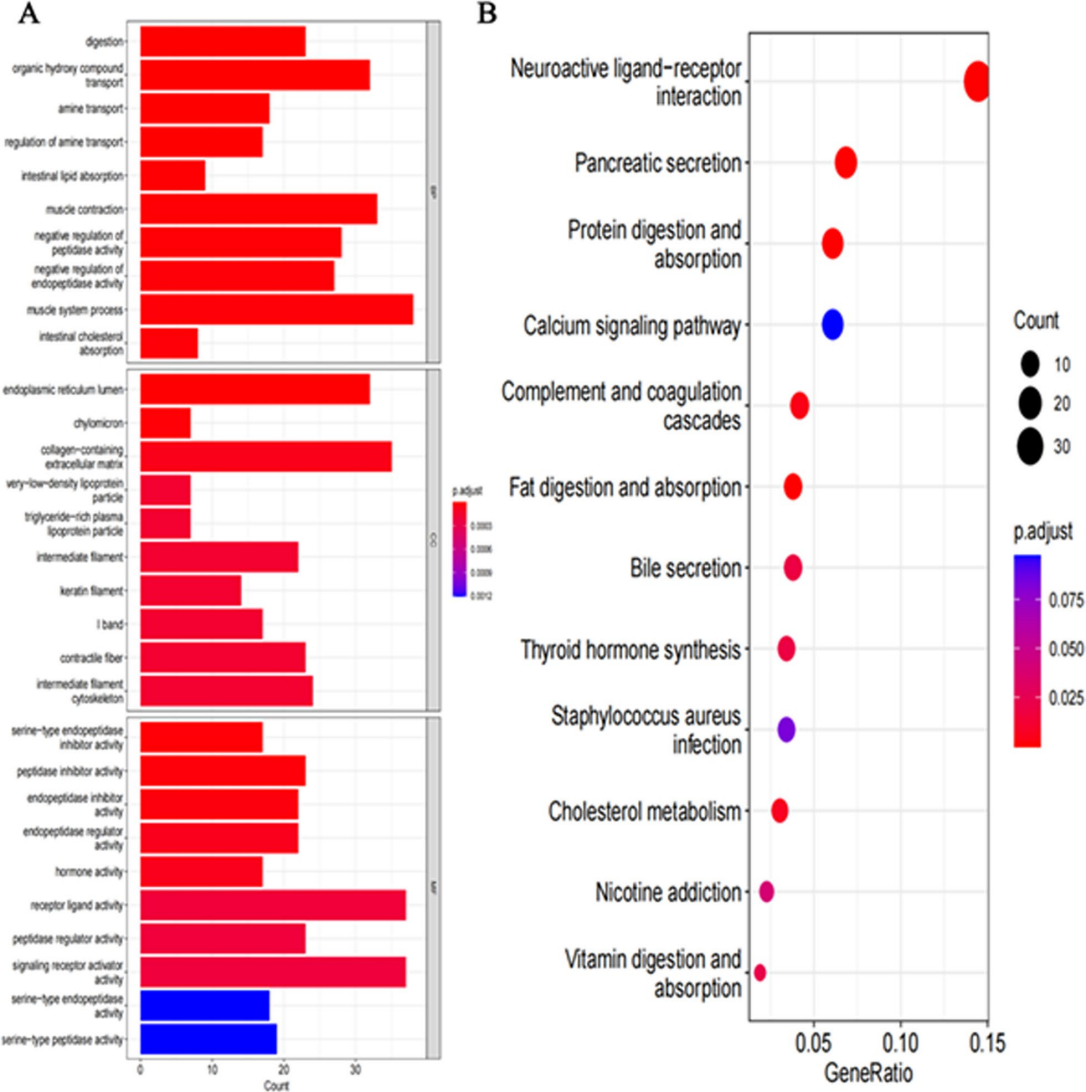
Enrichment analysis of CD44 by GO and KEGG. **A** Top 10 GO enrichment terms in BP, CC and MF in STAD. **B** The first 12 KEGG enrichment pathways in STAD

According to the data mined from TCGA, GO enrichment analysis was performed on the differential genes obtained from the analysis of CD44 divided into high and low groups. To explore the biological function and pathway of CD44, the differentially expressed genes with |log2FoldChange|> 1 and *P* < 0.05 were selected for enrichment analysis. The results showed the top 10 most significant biological effects under each item of biological process (BP), cell composition (CC) and molecular function (MF). The “count” on the horizontal axis represents the number of genes enriched in that item. The colour represents the corrected *P* value, and the redder the colour is, the stronger the difference. Analysis showed that CD44 was enriched in BP during digestion, absorption and transportation. These include digestion, organic hydroxy compound transport, amine transport and regulation, intestinal lipid absorption, etc. In CC, CD44 is predominantly located in the endoplasmic reticulum lumen, chylomicrons, and collagen-containing extracellular matrix. In MF, enrichment results suggest that CD44 is involved in the inhibition and regulation of various enzyme activities, such as serine-type endopeptidase inhibitor activity, endopeptidase inhibitor activity, and endopeptidase regulator activity (Fig. 4A).

In addition, bubble maps of the first 12 pathways of CD44 and its related genes were obtained by KEGG analysis [38–40]. The larger the bubble is, the redder it is and the greater the pathway is influenced by CD44 and related genes. It was found that the neuroactive ligand-receptor interaction was most associated with CD44. In addition, CD44 is closely related to the role of the digestive system in the body and the digestion and absorption of nutrients, including pancreatic secretion, protein digestion and absorption, and fat digestion and absorption (Fig. 4B).

### Gene set enrichment analysis (GSEA) of CD44-related signalling pathways

**Fig. 5:**
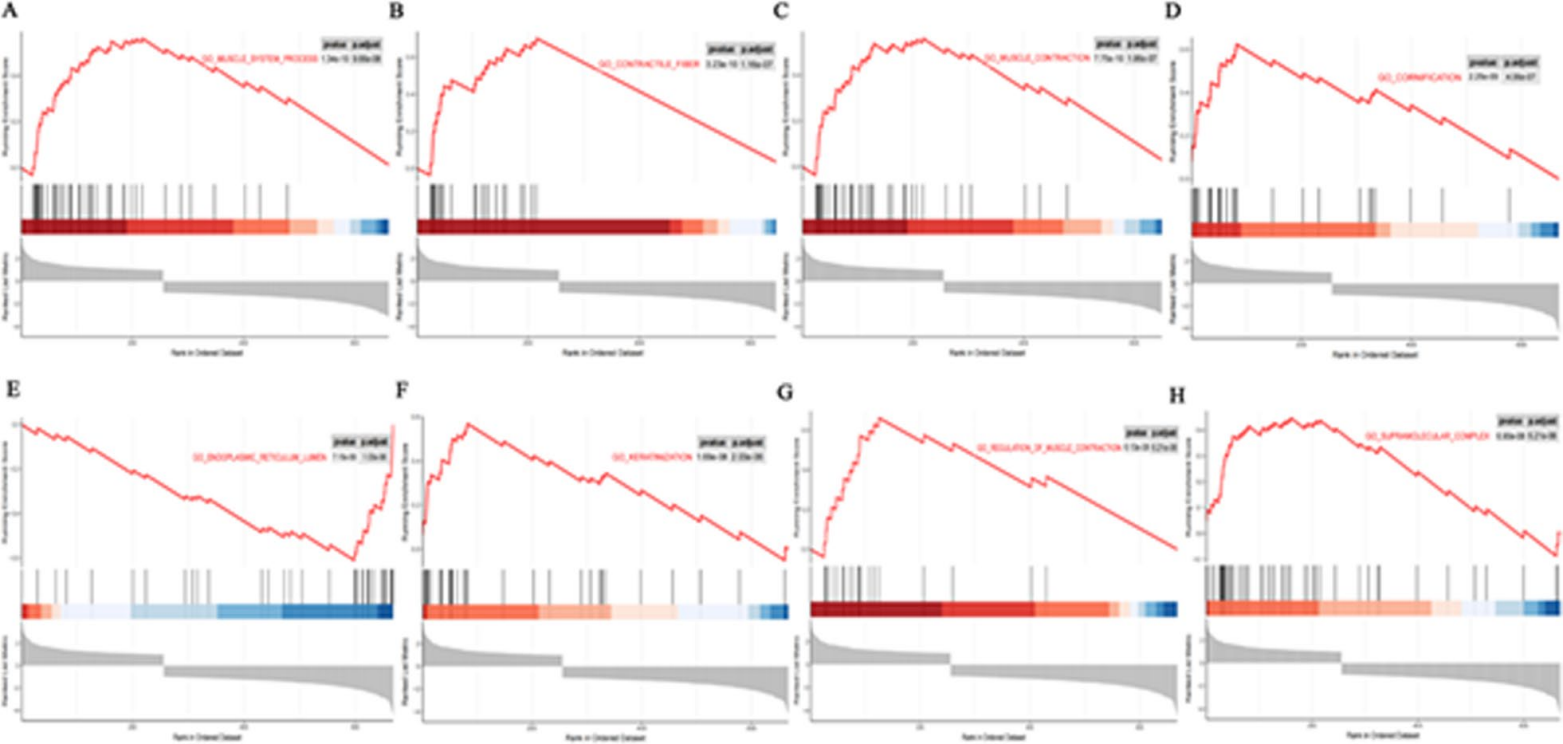
The pathways with the most CD44 participation were acquired through GSEA. **A**–**H** Including muscle system process, contractile fibre, muscle contraction, cornification, endoplasmic reticulum lumen, keratinization, regulation of muscle contraction, supramolecular complex, etc.

We used GSEA to further explore the specific molecular mechanism of CD44 in gastric cancer. After sequencing the genes in the gene set to be studied by multiple differences, a total of 117 signalling pathways were enriched. The results showed that the first eight pathways were enriched. CD44 is involved in muscle contraction regulation, epidermal cell differentiation and keratinization, endoplasmic reticulum lumen function, protein translation and modification, lipase activity regulation and so on. Among them, the enrichment score values of endoplasmic reticulum lumen pathways were all negative, indicating that the gene on the right side of the peak value was the core gene under the gene set (Fig. 5A–H). Because GSEA uses whole-genome expression profiles obtained by sequencing or chip analyses, there is no need to specify the threshold value of differential genes, and it can be used as a complement to GO and KEGG results.

### Relationship between infiltrating immune cells and CD44 expression

**Fig. 6:**
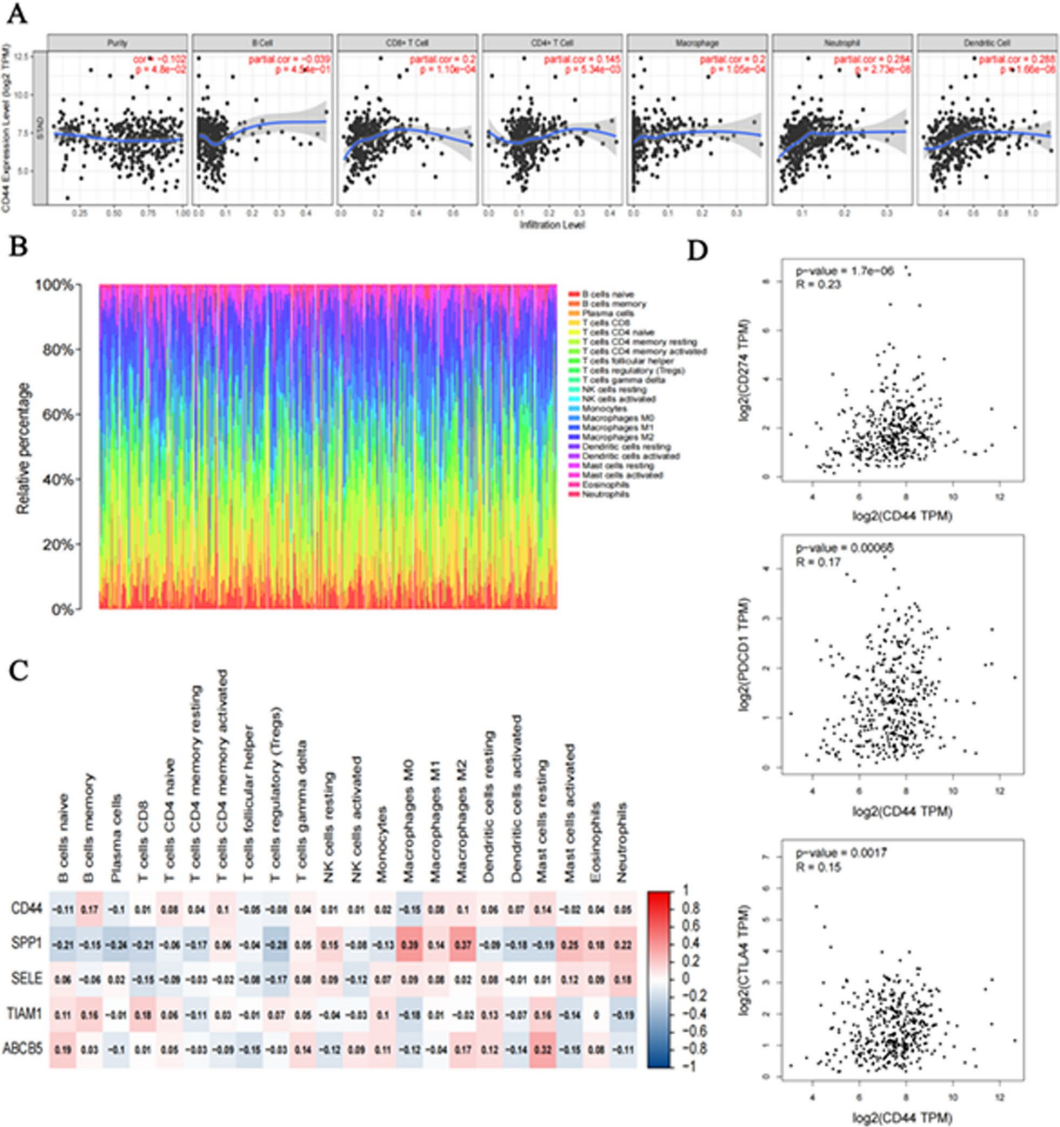
Correlation between CD44 expression and infiltrating immune cells. **A** Using the TIMER database, the correlation between the expression of CD44 in STAD and the infiltration of 6 kinds of immune cells was obtained. **B** The CIBERSORT algorithm was used to map 22 kinds of immune cells in tumour tissues of STAD patients. **C** A heatmap of the relationship between CD44 and a total of five genes associated with it and 22 immune cell types. **D** Scatterplot of the correlation between CD44 and CD274 and PDCD1 and CTLA4 obtained from the GEPIA database

There are not only tumour cells in the patient’s tumour tissue but also many immune cells infiltrates. By using the TIMER database, we analysed the correlation between the expression of CD44 and 6 kinds of infiltrating immune cells, namely, B cells, CD8+ T cells, CD4+ T cells, macrophages, neutrophils and dendritic cells, in gastric cancer. Analysis showed that CD44 was associated with CD8+ T cells, CD4+ T cells, macrophages, neutrophils and dendritic cells in patients with STAD. However, there was no significant correlation with B cells (Fig. 6A).

To further explore the invasion of tumour immune cells, CIBERSORT tumour immune microenvironment analysis was performed according to the expression profiles of STAD tumour patients obtained and sorted from the TCGA platform. After removing the immune cells that were all 0 in abundance, a map of 22 types of immune cells in tumour tissue was obtained. Each column represents a patient, and the sum of the 22 types of immune cells adds up to 100% (Fig. 6B). In addition, according to the CD44-related genes SPP1, TIAM1 and ABCB5 obtained in Fig. 3C–D, the correlation heatmap between them and infiltrating immune cells was drawn. The confidence interval was 0.95. Analysis showed that although the results in the TIMER database showed no correlation between CD44 and B cells, this conclusion was further refined in this result. That is, CD44 has a low correlation with native B cells, the highest correlation with memory B cells, and the lowest correlation with M0 macrophages. In addition, SPP1 is highly correlated with M0 macrophages and M2 macrophages, which can provide a direction for future research (Fig. 6C).

### Correlation analysis between CD44 and immune cell markers

The correlation between CD44 expression and various immune characteristics in STAD was verified by using the TIMER database. Table 1 lists 23 genes used to characterize B cells, T cells, CD8+ T cells, monocytes, M1 macrophages, M2 macrophages, neutrophils, natural killer cells and dendritic cells. Among them, tumour purity is a common confounding factor in tumour research. Therefore, tumour purity should be considered in the analysis of tumour immune-related genes. After adjusting for tumour purity, there was a correlation between CD44 outside CD8B and NOS2 and most of the immune markers (Table 1).

**Table 1:** The relationship between CD44 expression and immune gene markers in STAD was analysed by using the TIMER database

Description	Gene markers	STAD			
		None		Purity	
		Cor	*P*	Cor	*P*
B cell	CD19	0.157	**	0.137	**
	CD79A	0.164	***	0.137	**
T cell (general)	CD3D	0.229	***	0.205	***
	CD3E	0.224	***	0.199	***
	CD2	0.278	***	0.258	***
CD8+ T cell	CD8A	0.251	***	0.228	***
	CD8B	0.095	0.0528	0.078	0.1273
Monocyte	CD86	0.323	***	0.311	***
	CSF1R	0.291	***	0.280	***
TAM	CCL2	0.234	***	0.206	***
	CD68	0.242	***	0.229	***
	IL10	0.237	***	0.219	***
M1	IRF5	0.152	**	0.120	*
	PTGS2	0.170	***	0.146	**
	NOS2	0.010	0.8352	0.042	0.4132
M2	CD163	0.340	***	0.323	***
	VSIG4	0.231	***	0.228	***
	MS4A4A	0.313	***	0.304	***
Neutrophils	CEACAM8	0.091	0.0615	0.104	*
	ITGAM	0.361	***	0.343	***
	CCR7	0.246	***	0.344	***
Natural killer cell	KIR2DL1	0.199	***	0.205	***
Dendritic cell	HLA-DPB1	0.261	***	0.251	***

*P < 0.05, **P < 0.01, ***P < 0.001

In addition, we verified the correlation between CD44 and 13 functional T cells. After adjusting for tumour purity, CD44 was significantly correlated with 38T-cell markers in STAD (Table 2). To further verify the results in Table 2, Spearman’s correlation coefficient was selected for correlation analysis under the correlation plate of GEPIA. Scatter plots of the correlation of 3 T-cell immune checkpoints between CD44 and CD274 and between PDCD1 and CTLA4 were obtained. It was found that CD44 was significantly correlated with all three, which proved that CD44 plays an indispensable role in the immune microenvironment of gastric cancer (Fig. 6D).

**Table 2:** The correlation between CD44 and T-cell gene markers was analysed by using the TIMER database

Description	Gene markers	STAD			
		None		Purity	
		Cor	*P*	Cor	*P*
Th1	TBX21	0.275	***	0.256	***
	STAT4	0.275	***	0.273	***
	SATAT1	0.210	***	0.210	***
	TNF	0.167	***	0.127	*
	IFNG	0.183	***	0.165	**
Th1-like	HAVCR2	0.324	***	0.317	***
	IFNG	0.183	***	0.165	**
	CXCR3	0.203	***	0.193	***
	BHLHE40	0.237	***	0.231	***
	CD4	0.291	***	0.271	***
Th2	STAT6	0.333	***	0.338	***
	STAT5A	0.388	***	0.373	***
Treg	FOXP3	0.214	***	0.204	***
	CCR8	0.324	***	0.332	***
	TGFB1	0.268	***	0.251	***
Resting Treg	FOXP3	0.214	***	0.204	***
	IL2RA	0.344	***	0.333	***
Effector Treg T-cell	FOXP3	0.214	***	0.204	***
	CCR8	0.324	***	0.332	***
	TNFRSF9	0.287	***	0.294	***
Effector T-cell	CX3CR1	0.156	**	0.132	*
	FGFBP2	0.171	***	0.138	**
	FCGR3A	0.307	***	0.309	***
Native T-cell	CCR7	0.246	***	0.233	***
	SELL	0.330	***	0.313	***
Effector memory T-cell	DUSP4	0.119	*	0.117	*
	GZMK	0.252	***	0.227	***
	GZMA	0.255	***	0.227	***
Resident memory T-cell	CD69	0.323	***	0.304	***
	CXCR6	0.254	***	0.239	***
	MYADM	0.197	***	0.181	***
General	CCR7	0.246	***	0.233	***
Memory T-cell	SELL	0.330	***	0.313	***
	IL7R	0.339	***	0.323	***
Exhausted T-cell	HAVCR2	0.324	***	0.317	***
	LAG3	0.215	***	0.201	***
	CXCL13	0.253	***	0.251	***
	LAYN	0.299	***	0.286	***

### Correlation analysis of immune cell CD44 expression and survival prognosis of STAD patients

**Fig. 7:**
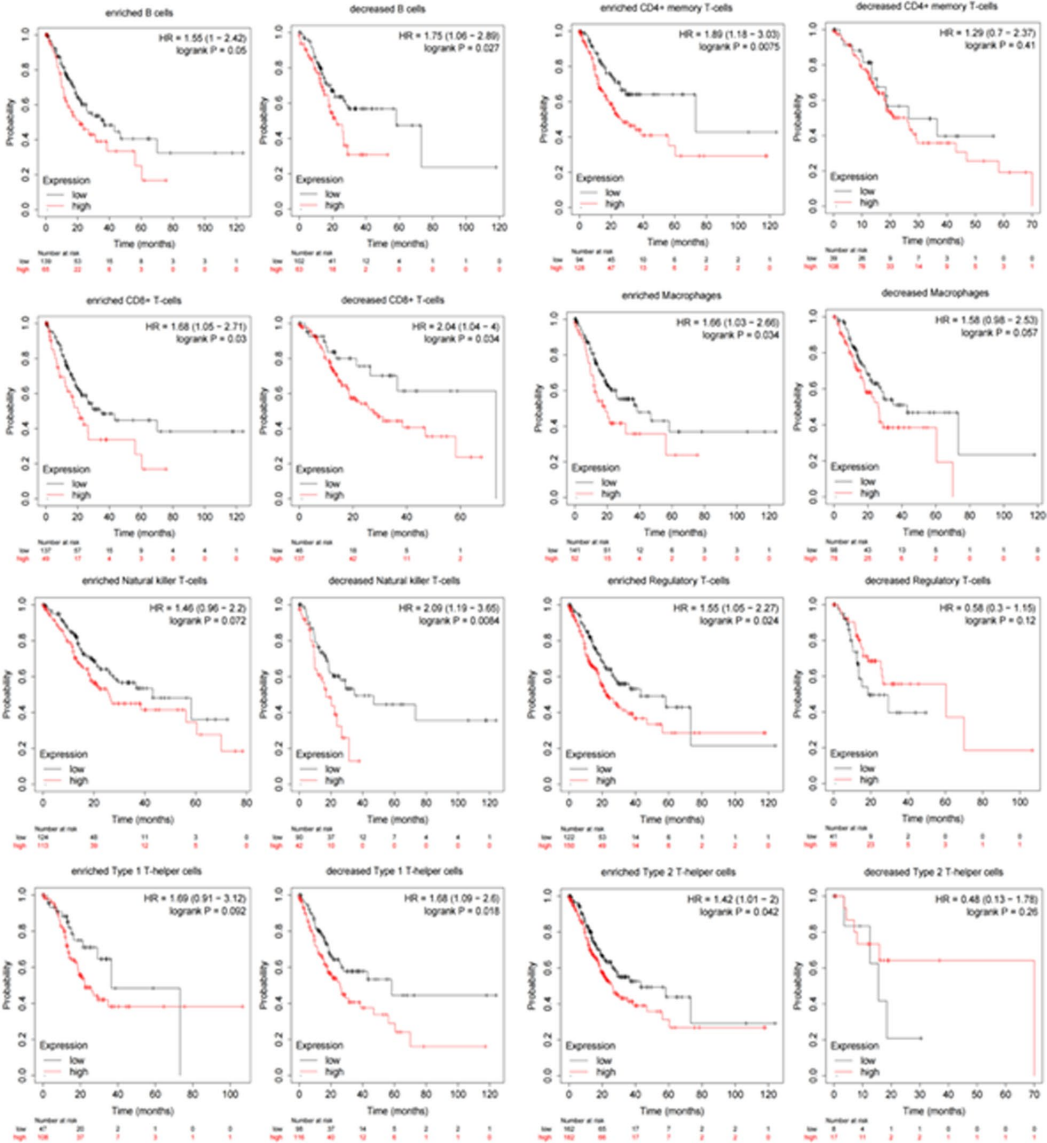
Kaplan‒Meier Plotter database analysis estimated the correlation between CD44 expression in 8 immune cell subpopulations and the survival prognosis of STAD patients

It can be seen from the above findings that the high expression of CD44 is closely related to the survival prognosis and immune cell infiltration of gastric cancer patients. We hypothesized that the survival prognosis of STAD patients was related to immune cell infiltration under the premise of high CD44 expression. To verify this hypothesis, pan-cancer RNA-seq based on the Kaplan‒Meier Plotter database was used to analyse the correlation between CD44 expression and prognosis in 8 immune cell subpopulations of gastric cancer. The results showed that under the premise of high expression of CD44, the prognosis of STAD patients was poor when the infiltration of B cells, CD8+ T cells, natural killer T cells and type 1T helper cells was reduced. However, there was no significant correlation between the prognosis of STAD patients and the expression of CD44 under the infiltration of CD4+ memory T cells, macrophages, regulatory T cells and type 2T-helper cells (Fig. 7). In conclusion, the expression of CD44 can affect the survival and prognosis of STAD patients through the infiltration of some immune cells.

## Discussion

Gastric cancer is a common malignant tumour of the digestive system caused by gastric mucosa [41, 41]. It has high morbidity and mortality. Because gastric epithelial cells need to be constantly renewed to meet the digestive function of the stomach, mutations in stem cells with high self-renewal, high proliferation and multi differentiation ability are often considered to be the aetiology of gastric cancer [42]. Patients with gastric cancer not only have tumour-related complications but also have a poor survival prognosis, which seriously affects human health. Because the symptoms of gastric cancer are very similar to those of gastric diseases such as gastritis and gastric ulcer, the serum indicators of gastric cancer, such as carbohydrate antigen 125 (CA125) and carbohydrate antigen 153 (CA153), are single and have poor sensitivity. Therefore, gastric cancer patients are often in the advanced stage when diagnosed, and the 5-year survival rate of patients with advanced gastric cancer is only 30%[43]. Based on this situation, with the progress of medical treatment, molecular targeted therapy may become the fourth treatment option [44]. Exploring markers related to gastric cancer invasion and metastasis may improve the early diagnosis rate and survival prognosis of gastric cancer.

In this study, we conducted bioinformatics analysis on the expression level of CD44 in gastric cancer tissues and normal tissues by using the TIMER, TCGA, GEO and HPA databases and found that the expression of CD44 in gastric cancer tissues was more than that in normal tissues, the results of the database analysis were also confirmed by comparing the mRNA expression in gastric cancer cells and normal cells (Fig. 1), In previous studies [17], CD44 expression in pan-cancer was obtained from the Oncomine database and TIMER database, while we used four databases for analysis, which were able to corroborate each other, and the results were more reliable and better guided by the difference in results after data update. Studies have proven that the positive expression rate of CD44 gradually increases with the progression of slow non atrophic gastritis, chronic atrophic gastritis, intestinal metaplasia, dysplasia, gastric cancer and other processes [45]. This suggests that CD44 can be regarded as an oncogenic gene that plays an important role in the early deterioration of gastric mucosa and thus can be used as a diagnostic criterion for precancerous lesions. The clinical prognostic significance of CD44 in patients with gastric cancer has not been reported through the UACLAN database, so we attempted and found that the expression of CD44 was not related to the patient’s age or sex but was related to TNM stage, tumour size, degree of invasion, degree of differentiation and lymph node metastasis. Kaplan‒Meier analysis showed that the survival rate of patients with high CD44 expression was significantly lower than that of normal subjects, this also validates the results previously reported in the literature [34]. Furthermore, cell proliferation and migration were reduced after the knockdown of CD44 expression in gastric cancer cells (Fig. 2). These results indicate that CD44 plays an important role in the staging, metastasis and prognosis of gastric cancer.

CD44 is a well-studied tumour stem cell marker, and it has been confirmed that there is an interaction between the opposite genes and CD44 (Fig. 3). Survivin, an inhibitor of apoptosis that can inhibit cell apoptosis and promote cell proliferation, is positively expressed in almost all tumour tissues [46]. It is positively correlated with CD44 and plays a synergistic role in the carcinogenesis of gastric cancer. SALL4 is a tumour stem cell marker found in acute myeloid leukaemia and is involved in the proliferation and migration of a variety of tumour cells [47]. Studies have confirmed that the expression of SALL4 and CD44 is also positively correlated in gastric cancer cells. SALL4 protein can directly promote the expression of CD44 by binding to the promoter region of CD44. This is an important factor promoting the migration of gastric cancer cells. The expression of miR-34a is downregulated in gastric cancer cells, and the increase in miR-34a is beneficial to gastric cancer patients. Experiments have shown that CD44, a downstream target gene of miR-34a, is negatively regulated by miR-34a [48]. miR-34a influences the survival prognosis of gastric cancer patients by regulating CD44. In addition, CD44, Wnt2, Bmi1, Notch1, Oct4, Sox2, Nanog, C-mys, ABCG2, CXCR4 and other “stemness associated genes” are highly expressed in the CD44+ gastric cancer cell population [49–52]. In conclusion, it is of great scientific value and clinical significance to explore targeted drugs that can specifically block the expression of CD44-interacting genes in future studies. In addition to the genes interacting with CD44, CD44 is involved in the regulation of multiple signalling pathways. GO enrichment showed that CD44 was involved in the formation of collagen-containing extracellular matrix and very low-density lipoprotein particles (Fig. 4A). Combined with the literature [12], it was concluded that CD44 is a class I transmembrane glycoprotein that can adhere to the extracellular matrix and bind to hyaluronic acid and collagen, playing an important role in the distant metastasis of tumours. In addition, CD44 can be used as a marker of the Hedgehog signalling pathway, in which SMO protein regulates GLI-1 nuclear translocation and promotes CD44 expression [53]. To improve the movement and survival of gastric cancer cells. In addition, highly expressed CD44 can activate the Wnt signalling pathway and promote the occurrence and metastasis of gastric cancer.

A key function of the immune system is to protect the organism against transformed tumours cells. Cytotoxic killer T lymphocytes (CTL) are a crucial cell type in this context as they are capable of directly destroying malignant cells, CD44 functions as a critical regulator of intra-tumoral movement by stabilizing cell polarity in migrating T cells [54]. Long-lived CD44+ tumour-initiating cells can selectively evade host immune responses and provide a rationale for targeting the PD-1 pathway in the adjuvant therapy setting of SCCHN [55]. Myeloid and tumour cell-expressed OPN and CD44 act as an immune checkpoint to suppress T cell activation and confer host tumour immune tolerance [56]. Programmed death-ligand 1 (PD-L1), also known as CD274, is a transmembrane protein that binds to the inhibitory receptor PD-1 on T cells and elicits T cell anergy, leading to immune suppression [57], Many cancer cells upregulate PD-L1 surface expression to escape immune surveillance [58–60]. CD44 could promote PD-L1 expression in breast and lung cancers [61], which suggests that it may indirectly affect the immune infiltration of tumours. In this study, we found that the expression of CD44 in gastric cancer was correlated with the infiltration degree of B memory cells, CD8+ T cells, CD4+ T cells, M1 macrophages, M2 macrophages, neutrophils, and dendritic cells (Fig. 6), similar analyses have been done in other tumours in the previous literature [17], but data are lacking in gastric cancer. In addition, CD44 affected the survival and prognosis of gastric cancer patients by affecting the infiltration of some immune cells (Fig. 7).

In conclusion, CD44 is expected to become a new immunotherapeutic target for gastric cancer by further exploration of the specific mechanism of CD44 and various immune cells and how it affect the survival and prognosis of gastric cancer patients.

This study systematically analysed the expression level of CD44 in gastric cancer, the clinical influencing factors of CD44 expression level, survival prognosis assessment, genes that interact with CD44, pathway enrichment, and the correlation between CD44 and the immune microenvironment through biogenic analysis. This study has further improved our understanding of the relationship between CD44 and gastric cancer. However, the specific mechanism of the interaction between CD44 and various immune cells, inflammatory factors and cytokines in the immune microenvironment needs further study. As a potential therapeutic prognostic marker of gastric cancer, CD44 has important research value and broad prospects.

## Conclusions

Our study further solidifies the key role of CD44 in gastric cancer and reveals its close relationship with prognosis and immune infiltration, suggesting that CD44 can be used as an exact target for the diagnosis and treatment of gastric cancer.

## Data Availability

The datasets analyzed during the current study are available in the following database, GEPIA2: http://portal.gbc.cancer.gov/, TCGA: https://portal.gdc.cancer.gov/, GEO: https://www.ncbi.nlm.nih.gov/geo/, HPA: https://www.proteinatlas.org, UALCAN: http://ualcan.path.uab.edu/index.html, Kaplan‒Meier Plotter: https://kmplot.com/analysis, Linked Omics: http://www.linkedomics.org/, GeneMANIA: http://genemania.org, STRING: https://string-db.org/, DisGeNET: https://www.disgenet.org/ (accession number:Houweiyan0519@163.com, TIMER: https://cistrome.shinyapps.io/timer/, GEPIA: http://gepia.cancer-pku.cn/.
